# A retrospective analysis of controlled active motion (CAM) versus modified Kleinert/Duran (modKD) rehabilitation protocol in flexor tendon repair (zones I and II) in a single center

**DOI:** 10.1007/s00402-022-04506-1

**Published:** 2022-08-17

**Authors:** C. Wirtz, F. M. Leclère, E. Oberfeld, F. Unglaub, E. Vögelin

**Affiliations:** 1 Department of Plastic and Hand Surgery, University of Bern, Inselspital, Freiburgstrasse, Bern, Switzerland; 2 Department of Hand Surgery, Vulpius Klinik, Vulpiusstraße 29, 74906 Bad Rappenau, Germany; 3 Medical Faculty Mannheim of the Ruprecht-Karls University Heidelberg, Theodor-Kutzer-Ufer 1-3, 68167 Mannheim, Germany

**Keywords:** Flexor tendon repair, CAM, Kleinert, Early active mobilization, Zone 2

## Abstract

**Introduction:**

The aim of this study was to analyze primary flexor tendon repair results in zones I and II, comparing the rupture rate and clinical outcomes of the controlled active motion (CAM) protocol with the modified Kleinert/Duran (mKD) protocol.

**Materials and methods:**

Patients who underwent surgery with traumatic flexor tendon lacerations in zones I and II were divided in three groups according to the type of rehabilitation protocol and period of management: group 1 included patients who underwent CAM rehabilitation protocol with six-strand Lim and Tsai suture after May 2014. Group 2 and 3 included patients treated by six-strand Lim Tsai suture followed by a modified Kleinert/Duran (modK/D) protocol with additional place and hold exercises between 2003 and 2005 (group 2) and between 2011 and 2013 (group 3).

**Results:**

Rupture rate was 4.7% at 12 weeks in group 1 (3/63 flexor tendon repairs) compared to 2% (1/51 flexor tendon repairs) in group 2 and 8% in group 3 (7/86 flexor tendon repairs). The grip strength at 12 weeks was significantly better in group 2 compared to the group 1 (35 kg/25 kg, *p* = 0.006). The TAM in group 1 [113° (30–175°)] was significantly worse (*p* < 0.001) than the TAM in group 2 [141° (90–195°)] but with similar extension deficits in both groups. The assessment of range of motion by the original Strickland classification system resulted in 20% excellent and 15% good outcomes in the CAM group 1 compared with 42% and 36% in the modK/D group 2. Subanalysis demonstrated improvement of good/excellent results according to Strickland from 45% at 3 months to 63.6% after 6-month follow-up in the CAM group.

**Conclusion:**

The gut feeling that lead to change in our rehabilitation protocol could be explained by the heterogenous bias. A precise outcome analysis of group 1 could underline that in patients with complex hand trauma, nerve reconstruction, oedema or early extension deficit, an even more intensive and individual rehabilitation has to be performed to achieve better TAM at 6 or 12 weeks. Our study explicitly demonstrated a significant better outcome in the modK/D group compared to CAM group. This monocenter study is limited by its retrospective nature and the low number of patients.

## Introduction

The functional results after flexor tendon repair in zones I and II remain a current topic of debate with regard to suture technique and the postoperative rehabilitation protocol. The dilemma of achieving a balance between reduction of scar formation without increasing risk of re-rupture is still unsolved. New developments in primary tendon repair in recent decades include stronger core tendon repair techniques, judicious and adequate venting of critical pulleys, followed by a combination of passive and active digital flexion and extension [1].

Different biomechanical studies have established that the strength of repair increases with the number of core sutures [2, 3]. The six-strand Lim and Tsai suture technique has shown a mechanical strength required for unrestricted active finger flexion in vitro [4, 5].

In an earlier publication, we demonstrated the benefit of a six-strand Lim Tsai suture followed by a modified Kleinert/Duran (modK/D) protocol with additional place and hold exercises over a two-strand suture technique combined with Kleinert/Duran rehabilitation alone [6].

For 7 years, the six-strand Lim/Tsai suture technique followed by the modK/D rehabilitation protocol was the standard treatment for flexor tendon repair in zone 1 and 2 in our clinic. After initial good results referring to rupture rate and range of motion (ROM) [6], an increase in the rate of secondary tendon rupture was noted in due course from 2011 to 2013. In this context, we questioned the use of another rehabilitation protocol to improve our results: the CAM rehabilitation protocol after flexor tendon repair was introduced by Small et al. [7] to improve postoperative range of motion by preventing restrictive adhesions.

The aim of this study was to clarify if the CAM protocol after primary flexor tendon repair in zones I and II lead to better outcomes compared to the modK/D protocol or if the gut feeling that lead to change in our surgical technique could be explained by heterogenous bias.

## Materials and methods

This clinical study was approved by our ethic committee (KEK: 2017-02095). Clinical and functional outcome from patients who underwent surgery with traumatic flexor tendon lacerations in zones I and II were assessed retrospectively. Inclusion criteria and exclusion criteria are reported in Table 1. Patients were divided in three groups according to the type of rehabilitation protocol and period of management: group 1 included patients who underwent CAM rehabilitation protocol after six-strand Lim and Tsai suture (Table 2). Group 2 and 3 included, respectively, patients treated by six-strand Lim Tsai suture followed by a modified Kleinert/Duran (modK/D) protocol with additional place and hold exercises between 2003 and 2005 [6] and between 2011 and 2013 (Table 2).

**Table 1:** Inclusion/exclusion criteria in our study

Inclusion criteria	Exclusion criteria
Treatment within 7 days	Bone, joint and severe skin damage requiring additional surgery
Postoperative therapy for a minimum of 8 weeks	Age < 13 years
Follow-up min 12 weeks	Replantation/revascularization
Recording of age, gender and details of injury	Rehabilitation other than CAM
	Suture technique other than Lim/Tsai
	No data or loss to follow-up

*CAM* controlled active motion

**Table 2:** The three groups of patients in ours study according to the type of rehabilitation protocol and period of inclusion

	Group 1 CAM protocol After May 2014	Group 2 Modified Kleinert/Duran protocol 2003–2005	Group 3 Modified Kleinert/Duran protocol 2011–2013
Suture technique	Lim Tsai (six strand) + epitendinous suture	Lim Tsai (six strand) + epitendinous suture	Lim Tsai (six strand) + epitendinous suture
Suture material	Supramid 4.0 + Prolene 6.0	Supramid 4.0 + Prolene 6.0 or 5.0	Supramid 4.0 + Prolene 6.0 or 5.0
Postoperative management	Active extension, passive flexion followed by controlled active flexion (without place and hold)	Active extension, passive flexion, with place and hold	Active extension, passive flexion, with place and hold
	Dorsal forearm orthosis wrist 30° extension MCP 30° flexion IP 0°	Dorsal forearm orthosis with rubber-band traction to injured digits wrist 30° flexion MCP an IP allowed full active extension to 0°	Dorsal forearm orthosis with rubber-band traction to injured digits wrist 30° flexion MCP an IP allowed full active extension to 0°
	Orthosis for 6 weeks day and night until the 8th week just at night	Orthosis for 3.5 weeks day and night + 1.5 weeks with a simple wrist cuff rubber-band extension	Orthosis for 3.5 weeks day and night + 1.5 weeks with a simple wrist cuff rubber-band extension
Patients	56 (11 female 19.6%, 45 male 80.4%)	46 (8 female 17.4%, 38 male 82.6%)	48 (15 female 31.2%, 33 male 68.8%)
Age (range min–max)	37 (18–74)	32 (13–74)	37.8 (13–78)
Injured hand			
Dominant	21	22	26
Nondominant	35	24	22
Injured digits	63	51	60
	17 index 14 middle 12 ring 20 little	13 index 9 middle 7 ring 22 little	13 index 12 middle 10 ring 25 little
Injured tendon			
Isolated FDP	28 (44%)	24 (47%)	18 (30%)
FDP complete, FDS partial	11 (17%)	9 (17%)	15 (25%)
Complete FDP and FDS	22 (31%)		
Neurovascular injury	33	36	30

*CAM* controlled active motion, *modKD* modified Kleinert/Duran (modKD) rehabilitation

### Surgical technique of tendon repair in all groups (Figs. 1, 2)

**Fig. 1:**
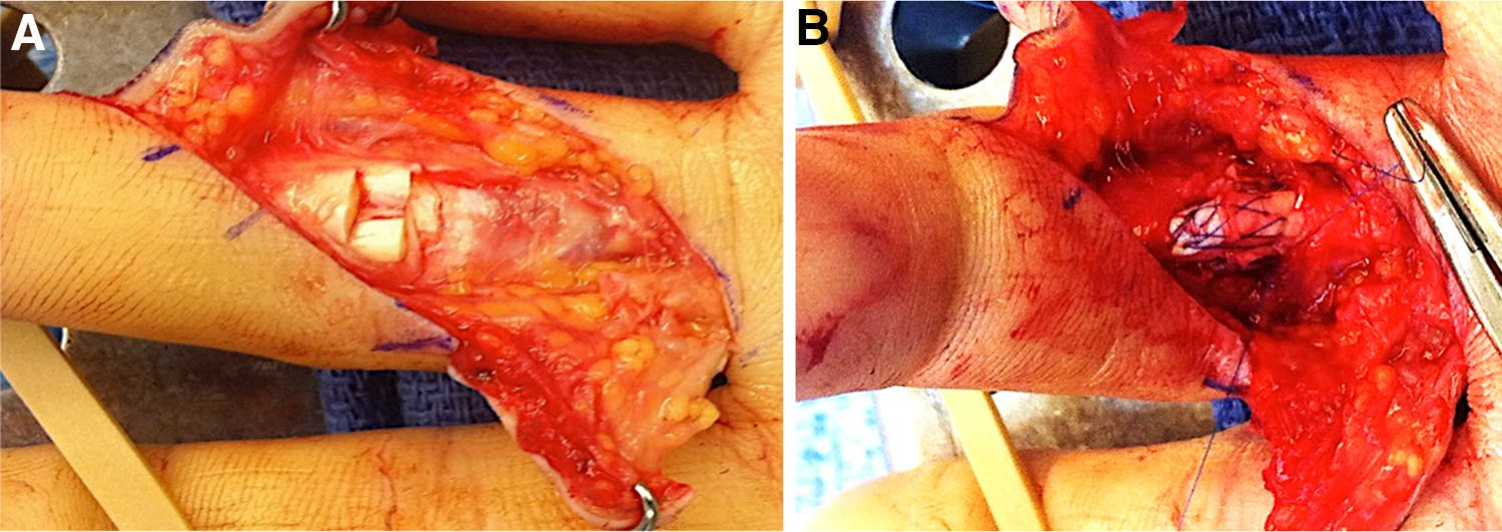
Before (**A**) and after (**B**) six-strand Lim/Tsai suture technique for tendon repair

**Fig. 2:**
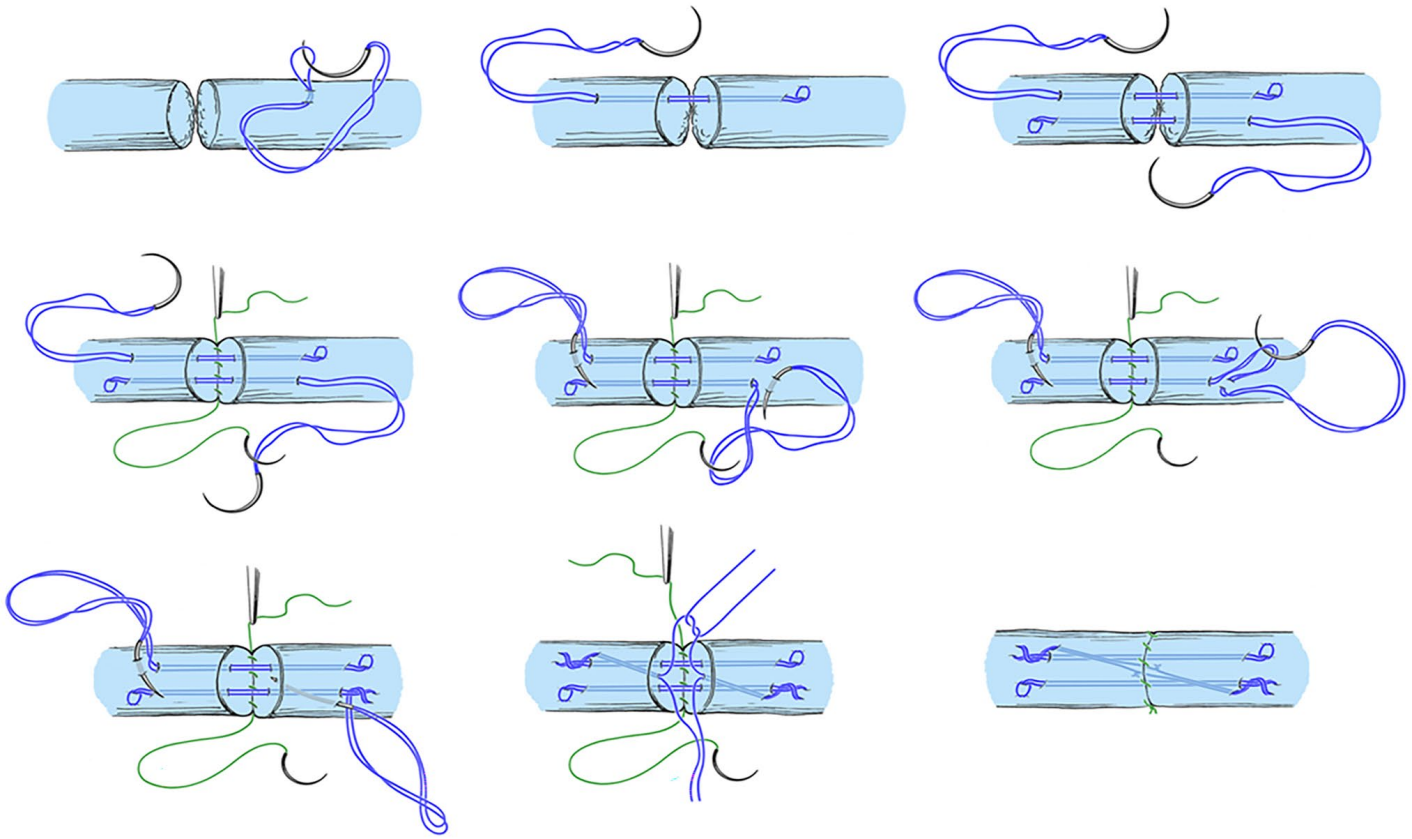
The six-strand Lim/Tsai suture technique

As described by Lim and Tsai [4], the deep flexor tendons were repaired using a 6-0 strand core suture with locking loops. Suture material was 4–0 or 3–0 polyester braid containing a long chain polyethylene core Supramid (ERMED AG, Schleitheim, Germany). All sutured tendons were repaired using additional circumferential epitendinous suture as described by Silverskijöld with 6-0 polypropylene Prolene 5-0 or 6-0 (Johnson & Johnson Medical, New Brunswick, NJ). Before wound closure, free gliding of the tendon under the pulleys and gapping at the repair site were tested, performing full extension/flexion of all joints, described as the extension-flexion test by Tang [8]. Venting of the annular pulleys was performed if indicated. In some cases, pulley repair was performed.

### Postoperative rehabilitation in group 1: CAM protocol (Table 2)

**Fig. 3:**
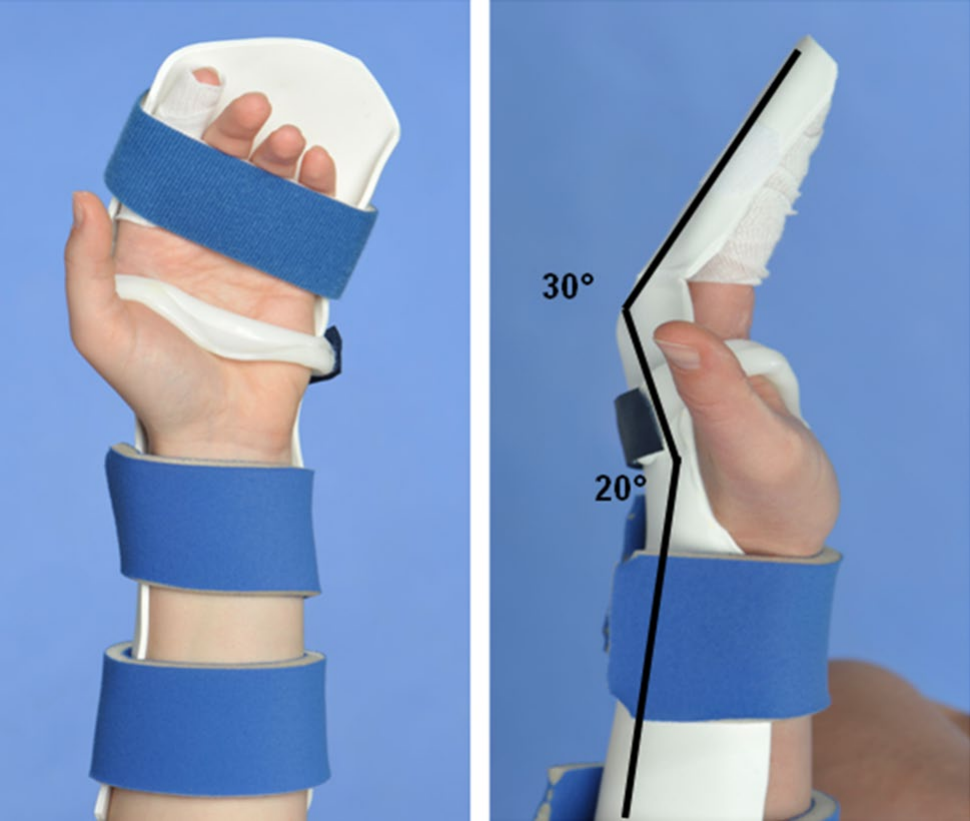
Splint for CAM protocol in group 1

The CAM protocol was used since 2014. A dorsal forearm-based thermoplastic orthosis with the wrist in 20–30° of extension, the MCP joints in 30° flexion and the IP joints in 0° extension was applied by the hand therapists within 3–5 days after surgery (Fig. 3). The orthosis was worn day and night for 6 weeks and only at night until the 8th week. Tenodesis exercises outside the orthosis were allowed from the third postoperative week. Active motion of the fingers was initiated at the day of application of the thermoplastic orthosis, five times per day. Home exercises started with full passive mobilization (depending on the extent of postoperative swelling) followed by active flexion, which had to be initiated from the DIP joint to maximize differential glide. Full active finger flexion was allowed in a staged program until the 4th week. The patients were encouraged to perform active digital extension exercise to minimize the risk of interphalangeal joint flexion contractures. Any residual flexion contractures were treated with finger-based extension splints. Patients continued to exercise active flexion and extension, tenodesis exercises were started in the 4th week and blocking exercises in the 6th week. Loading exercises and light activities of daily living were initiated in the 8th week and full use was permitted after 12 weeks. The patients were seen weekly in our hand therapy.

### Postoperative rehabilitation in groups 2 and 3 (Table 2): modK/D protocol (Fig. 4)

**Fig. 4:**
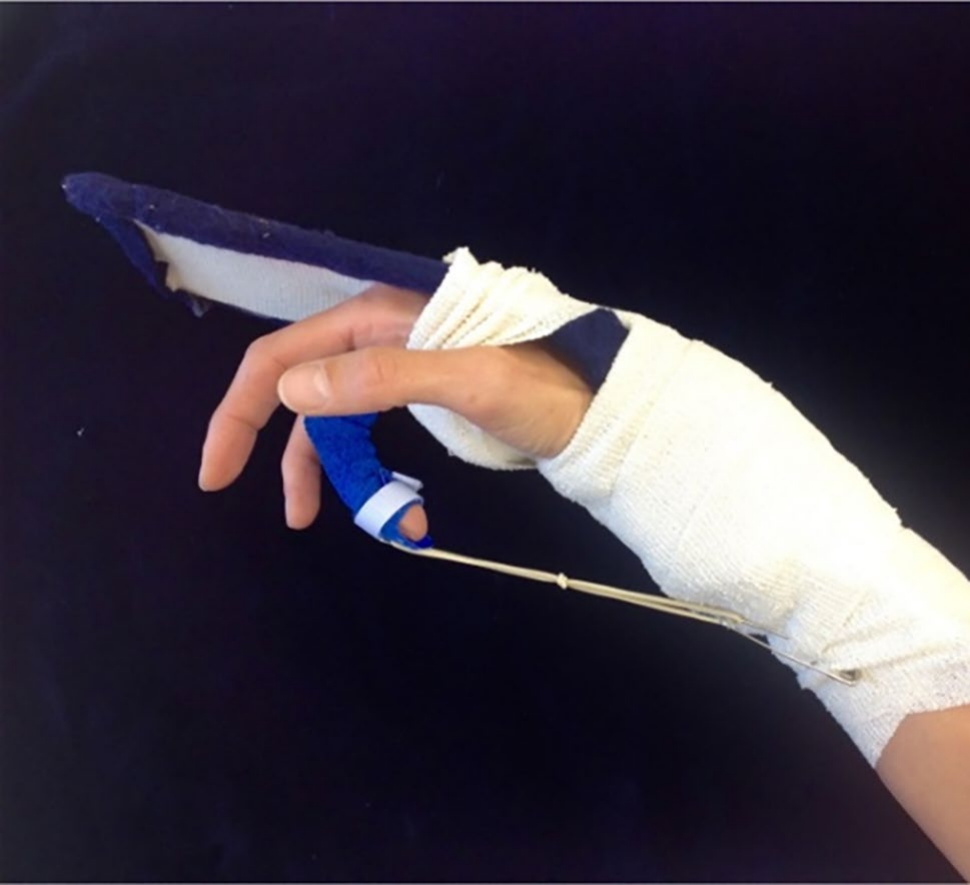
Splint for modK/D protocol in group 2 and group 3

The modK/D protocol consisted of the following rehabilitation: in addition to the Kleinert/Duran regime, place and hold exercises [9] were done for 5 weeks, starting on the first postoperative day (Fig. 4). Our modified Kleinert/Duran regime [6] included 3.5 weeks use of a dorsal blocking orthosis with rubber-band traction to the injured digits, 1.5 weeks with a simple wrist cuff rubber-band assembly, followed by active mobilization. The position of the wrist in the orthosis was 30° short of maximal flexion, the metacarpophalangeal (MP) and interphalangeal joints (PIP and DIP) of the fingers and thumb being allowed full active extension. Place and hold was carried out with dorsal splint protection during the first 3.5 weeks three times a day. The recommended frequency of the rubber-band-assisted passive flexion–active extension exercises was six to eight times a day.

### Functional assessment

At the 3-month postoperative control, the flexor tendons were tested separately to assess re-rupture. Grip strength measurement was made with a dynamometer (Jamar, Boling Brook, IL). The original Strickland grading system was used, to assess final total active motion (TAM) [10] (Table 3). The functional results were recorded after 6 and 12 weeks postoperatively in the CAM group. In the modK/D group, the assessment was at 12 weeks after surgery.

**Table 3:** Strickland classification used in our study

Excellent	> 149**°**
Good	125–149°
Fair	90–124°
Poor	< 90°

### Statistical analysis

Outcomes in the three groups were compared using linear regression with robust standard errors. All estimated differences between the groups are accompanied by 95% confidence intervals and *p* values testing the null hypothesis that there is no difference between the groups. Due to heterogenicity of group 3, it was not possible to make a fine statistical analysis in this group.

## Results

The results of the three groups are summarized in Table 2. Gender as well as age distribution was similar in the groups (*p* < 0.001). In the CAM group, one patient was lost to follow-up at 12 weeks. Due to change in surgeons, therapists and patient education and compliance, group 3 was heterogenous and it was not possible to make any statistical analysis in this group.

### Rupture rate

The rupture rate in group 1 was 4.76% (3/63) compared to 2% (1/51) in group 2 and 8.14% (7/86) in group 3 (Table 4). In the CAM group (group 1), this included two patients in which a venting of the A5 and/or the A4 pulley was performed intraoperatively. In one patient, there was a delay of the tendon repair of 7 days as the only noticeable parameter. The results of the other analyzed parameters did not vary with patients without re-rupture. Re-ruptures occurred 2× at 1 week, and the other one at 8 weeks. In group 3, there were seven re-ruptures in four patients (8.14%), all men, age mean 43.75 years (25–74 years). Four re-ruptures were seen in the same patient in different fingers (II, III, IV, and V). In the other three patients, index, middle and small fingers were involved. Re-ruptures occurred 4× at 6 weeks, 2× at 8 weeks and once at 12 weeks.

**Table 4:** Outcomes at 12 weeks in our study

Parameters	CAM mean (SD; min–max)	mK/D mean (SD; min–max)	Mean difference (95% CI^a^; *p* value)
Total active motion (TAM)			
PIP + DIP	113° (30–175°)	141° (90–195°)	
MCP + PIP + DIP	201° (37.2°; 96.0–275°)	233° (25.7°; 187–279°)	− 31.95 (− 45.25 to − 18.65; < 0.001)
Extension deficit (ED)			
PIP + DIP	14.0° (13.6; 0.000–50.0°)	12.2° (6.43; 0.817–23.6°)	1.83 (− 2.44 to 6.10; 0.397)
Grip strength			
Injured hand	25.3 kg (8–52 kg)	34.6 kg (14–60 kg)	
Uninjured hand	43 kg (16–73 kg)	45 kg (22–70 kg)	
Differences uninjured injured	17.7 kg (9.48; 2.00–47.3 kg)	11.0 kg (20.6; − 25.4 to 47.6 kg)	6.70 (− 0.46 to 13.86; 0.066)
Rupture rate	3/63 (4.76%)	1/51 (2%) 7/86 (8.14%)^b^	

*CAM* controlled active motion, *modKD* modified Kleinert/Duran (modKD) rehabilitation

^a^Confidence interval

^b^Rupture rate in group 3

### Grip strength (Table 4)

The grip strength at 12 weeks was significantly better (*p* = 0.006) in group 2 (modK/D) (34.6 kg injured hand, 45 kg uninjured hand) compared to the CAM group (25.3 kg injured hand, 43 kg uninjured hand).

### Total active motion (TAM) and extension deficit (ED) at 12 weeks (Table 4)

Due to reasons previously explained in this article, it was only possible to compare group 1 with group 2. The TAM in the CAM group [113° (30–175°)] was worse (*p* < 0.001) than the TAM in the mK/D group [141° (90–195°)]. The average extension deficit was similar in both groups with 13° (CAM group) and 12° (mK/D group), on average 1.83° worse in the CAM group. The assessment of range of motion by the original Strickland classification system (Table 3) resulted in 20% excellent and 15% good outcomes in the CAM group compared with 42% and 36% in the mK/D group (Table 5). Regarding the CAM group, in the poor/fair group (*n* = 38), there were five cases of CRPS and one case of postoperative infection (6/38) compared to one case of CRPS (1/21) in the good/excellent group (Table 6).

**Table 5:** Results at 12 weeks/6 months assessed by the original Strickland system

	CAM		mK/D
	12 weeks	6 months	12 weeks
Excellent	12/59 (20%)	17/54 (31%)	21/50 (42%)
Good	9/59 (15%)	13/54 (24%)	18/50 (36%)
Fair	27/59 (46%)	16/54 (30%)	–
Poor	11/59 (18%)	8/54 (15%)	11/50 (22%)

*CAM* controlled active motion, *modKD* modified Kleinert/Duran (modKD) rehabilitation

**Table 6:** Analysis of influencing factors within the CAM group according to Strickland classification

Strickland system	Excellent/good CAM *n* = 21	Poor/fair CAM *n* = 38
Age	21 (18–29 y)	43 (24–68 y)
Gender ratio: female:male	1:9.5	1:3,2
	(2f, 19m)	(9f, 29m)
Dominance		
Nondominant:dominant	1:1.3	1:1.9
	Nondominant: 57% (*n* = 12)	Nondominant: 34% (*n* = 13)
	Dominant: 43% (*n* = 9)	Dominant: 66% (*n* = 25)
Zone of injury	Zone 1: 19% (*n* = 4)	Zone 1: 13% (*n* = 5)
	Zone 2: 81% (*n* = 17)	Zone 2: 87% (*n* = 33)
Mechanism of injury	1 dull	1 dull
	20 sharp	37 sharp
Concomitant injury (nerve/vessel)	43% (9/21)	39% (15/38)
Pulley injury or venting	57.5% (12/21)	42% (16/38)
Finger	Dig II: 6	Dig II: 11
	Dig III: 6	Dig III: 6
	Dig IV: 3	Dig IV: 8
	Dig V: 6	Dig V: 13
Time to surgery (d)	Mean 2 d (0–6 d)	Mean 1.1 d (0–4 d)
Pluridigital injuries	9.5% (2/21)	18% (7/38)
CRPS	4.7% (1/21)	13% (5/38)
Infection	0% (0/21)	3% (1/38)
FDS tendon repair	66.7% (6/9)	40% (10/25)
No FDS tendon repair or resection	33.3% (3/9)	56% (14/25)

*CAM* controlled active motion

## Discussion

The aim of this study was to clarify if the CAM protocol in flexor tendon repair (zone I and II) lead to better outcomes compared to the modK/D protocol or if the gut feeling that lead to change in our surgical technique could be explained by the heterogenous bias. Rupture rate was 4.7% at 12 weeks in group 1 (3/63 flexor tendon repairs) compared to 2% (1/51 flexor tendon repairs) in group 2 and 8% in group 3 (7/86 flexor tendon repairs). The TAM in group 1 (113°) was significantly worse than the TAM in group 2 (141°) but with similar extension deficits in group 1 and 2. The assessment of range of motion by the original Strickland classification system resulted in 20% excellent and 15% good outcomes in the CAM group 1 compared with 42% and 36% in the modK/D group 2.

Until now, no single early active motion protocol has been proven to be the ‘‘gold standard’’ for flexor tendon rehabilitation. Each was developed in a different clinical setting, with different surgical techniques and different patient groups [11]. In a systematic review of different flexor tendon repair rehabilitation protocols, Starr et al. [12] showed a statistically significantly higher risk of decreased digit range of motion (defined as extension lag > 15° or joint contracture of 20°) of 9% but lower rupture rate of 4% in the passive rehabilitation protocols compared to higher risk of tendon rupture (5%) but better postoperative digit range of motion (6%) in early active motion protocols.

After initial good results with the modified Kleinert/Duran (modKD) Rehabilitation Protocol in flexor tendon repair referring to rupture rate and range of motion (ROM) [6], an increased in the rate of secondary tendon rupture was noted in due course. In this context, our rehabilitation protocol was readapted to reduce re-rupture rate and improve tendon excursion: (i) eliminate “place and hold” exercises to reduce tension to tendon suture during exercises, (ii) improve tendon gliding by wrist positioning in 20° extension at day 3–5. With this CAM protocol, tendon excursion is increased by the addition of wrist tenodesis. There is support for a tenodesis pattern that combines MCP extension with wrist extension and PIP joint flexion to promote greater tendon excursion at the FDP tendon [13]. This study aimed at comparing the clinical outcomes after our usual rehabilitation protocol with this new protocol of rehabilitation with a special focus on rupture rate and the range of motion.

(i) Our study could conclude that both adaptions are associated with significant lower range of motion 3 months after surgery in the CAM group compared to modK/D group. We cannot conclude, weather elimination of “place and hold” exercises or changing of wrist position in the splint led to lower range of motions or if it is the combination of both.

(ii) The rupture rate of 4.76% in the CAM group 1 is comparable with the 5% rupture rate in most early active motion protocols [12]. In other words, the rupture rate is not better with this new protocol. In the CAM group, there were two re-ruptures, in one patient after 10 days without wearing the brace and use of his operated hand without limitations. The patient refused further treatment. A second patient showed one re-rupture of the little finger in zone 2 of the dominant hand after 8 weeks. No adequate trauma or special condition was obvious in his postoperative course that could explain re-rupture, except a delay of 7 days until the primary repair. Two staged tendon reconstruction was performed in due course. In the mK/D groups, the rupture rate increased from 2% (group 2) [6] to 8.14% (group 3) in our patients and is higher than the rupture rate in other early active motion protocols [1, 12]. A detailed analysis of group 3 showed seven re-ruptures in four patients: four re-ruptures occurred in the same patient. Two staged flexor tendon repair of the FDP II–V was performed afterwards and secondary tendon re-rupture occurred again in all tendon grafts. In this clinical case, there was a problem with malcompliance, nicotine abuse and the diagnosis of hypermobility syndrome (ICD M35.7). Although these factors are not known risk factors for secondary tendon rupture, this is a special case with unusual complications due to external factors, that negatively influenced the statistical result of this group. In other words, the rupture rate would be 3.49% without this patient. This is better than the 5% rupture rate in most early active motion protocols [12]. Reasons for re-rupture in the 3 other repairs were one adequate trauma (fall in the shower) in one case and no obvious reason in the other two patients.

(iii) The high percentage of poor or fair outcomes at the 3-months follow-up in the CAM group might be due to a conservative and limited active range of motion in the CAM rehabilitation protocol. On the other hand, the high percentage of poor and fair results is certainly affected by the high rate of complications such as CRPS (*n* = 5), postoperative infection (*n* = 1) and pluridigital injury pattern (*n* = 7). All these factors are known to be associated with worse functional results after flexor tendon repair [14]. However, without these cases, the rate of poor or fair results is still 39% and remains higher than in most reports [15–18]. Other factors, that were noticed in the subanalysis of group 1 were: A2 or A4 pulley reconstruction (*n* = 3), dystrophy (*n* = 2), nerve reconstruction with allograft (*n* = 1), lymphatic edema (*n* = 1) or neuroma (*n* = 1). Rigo and Rokkum [14] have well demonstrated that these factors are also known to be associated with poor outcomes. Giesen [15] mentioned the role of edema in complex hand finger trauma: movement of edema onto the dorsum of the hand carries fibrin with it, and restricts also the movement of the digits into flexion.

This pathology is probably a greater cause of morbidity after flexor tendon surgery, wherever and, however, the repair is done and whoever does the surgery. Anti-edema bandage to fingers and the hand immediately after the repair may help to reduce edema and avoid later adhesions with fibrin.

Despite reassuring results on rupture rate in the three groups of patients and precise analysis of the CAM protocol outcomes, these study present two limitations: first, it was not possible to make a statistical analysis in group 3 due to heterogeneous reasons. Moreover, it was a monocenter retrospective study limited by its number of patients.

## Conclusion

The gut feeling that lead to change in our rehabilitation protocol could be explained by the heterogenous bias. A precise outcome analysis of group 1 could underline that in patients with complex hand trauma, nerve reconstruction, oedema or early extension deficit, an even more intensive and individual rehabilitation has to be performed to achieve better TAM at 6 or 12 weeks. Our study explicitly demonstrated a significant better outcomes in the modK/D group compared to CAM group. This monocenter study is limited by its retrospective nature and the low number of patients.
